# Prevalence of livestock associated MRSA in blood isolates

**DOI:** 10.1186/1753-6561-5-S6-P170

**Published:** 2011-06-29

**Authors:** BV Cleef, BV Benthem, J Monen, A Haenen, NVD Sande-Bruinsma, J Kluytmans

**Affiliations:** 1 Centre for Infectious Disease Control Netherlands, RIVM National Institute for Public Health and the Environment, Bilthoven, Netherlands; 2 Department of Medical Microbiology and Infection Prevention, VU University Medical Centre, Amsterdam, Netherlands

## Introduction / objectives

In the Netherlands there is an extensive reservoir of livestock associated methicillin-resistant *Staphylococcus aureus* (LA-MRSA) in pigs and calves. The aim of this study was to establish the prevalence of LA-MRSA in human blood isolates.

## Methods

This study was based on data from the national antibiotic resistance surveillance (ISIS-AR) in The Netherlands. The 22 participating laboratories cover approximately 50% of all hospital beds. Data from 2008 through 2010 on *S. aureus* (SA) isolates were evaluated for methicillin resistance and *spa*-type, to identify LA-MRSA strains. Only the first isolate per patient was included. For this preliminary examination, we used tetracycline resistance as an indicator for LA-MRSA. In further analysis presented at the congress, *spa*-types will be included.

## Results

The proportion of MRSA of all episodes of SA bacteremia was 1.5% (51/3355). Of the MRSA isolates with an antibiotic resistance profile, 17% were tetracycline resistant (8/48, 95%CI 9-30%). The proportion of tetracycline resistance in MRSA isolates from other sources was 41% (872/2124, 95%CI 39-43%, chi-square blood vs non-blood p<0.0001). Extrapolation results in an average annual incidence of 5.3 patients with LA-MRSA bacteremia in The Netherlands.

## Conclusion

The current annual incidence of LA-MRSA bacteremia is low. Tetracycline resistant MRSA is significantly less prevalent in MRSA blood isolates compared to non-blood isolates. This can be the result of an excessive screening regime resulting in overrepresentation in non-invasive isolates or a decreased virulence of the livestock associated strains.

## Disclosure of interest

None declared.

